# The Influence of Lactic Acid Fermentation on Selected Properties of Pickled Red, Yellow, and Green Bell Peppers

**DOI:** 10.3390/molecules27238637

**Published:** 2022-12-06

**Authors:** Emilia Janiszewska-Turak, Dorota Witrowa-Rajchert, Katarzyna Rybak, Joanna Rolof, Katarzyna Pobiega, Łukasz Woźniak, Anna Gramza-Michałowska

**Affiliations:** 1 Department of Food Engineering and Process Management, Institute of Food Sciences, Warsaw University of Life Sciences—SGGW, 02-787 Warsaw, Poland; 2 Department of Food Biotechnology and Microbiology, Institute of Food Sciences, Warsaw University of Life Sciences—SGGW, 02-787 Warsaw, Poland; 3 Department of Food Safety and Chemical Analysis, Institute of Agricultural and Food Biotechnology, 36 Rakowiecka Street, 02-532 Warsaw, Poland; 4 Department of Gastronomy Science and Functional Foods, Faculty of Food Science and Nutrition, Poznań University of Life Sciences, Wojska Polskiego 31, 60-624 Poznań, Poland

**Keywords:** bell peppers, texture, chlorophylls, carotenoids, antioxidant properties, sugars, LAB

## Abstract

Red, yellow, and green peppers are vegetables rich in natural pigments. However, they belong to seasonal vegetables and need to be treated to prolong their shelf life. One new approach to processing vegetables is to pickle them using lactic acid bacteria. The use of such a process creates a new product with high health value, thanks to the active ingredients and lactic acid bacteria. Therefore, this study aimed to evaluate the effect of the applied strain of lactic acid bacteria (LAB) on the chemical properties, including the content of active compounds (pigments) and the physical properties of the peppers. *Levilactobacillus brevis*, *Limosilactobacillus fermentum*, and *Lactoplantibacillus plantarum* were used for fermentation and spontaneous fermentation. The pigments, polyphenols content, and antioxidant properties were determined in the pickled peppers, as well as sugar content, color, dry matter, texture properties, and the count of lactic acid bacteria. In all samples, similar growth of LAB was observed. Significant degradation of chlorophylls into pheophytins was observed after the fermentation process. No significant differences were observed in the parameters tested, depending on the addition of dedicated LAB strains. After the fermentation process, the vitamin C and total polyphenols content is what influenced the antioxidant activity of the samples. It can be stated that the fermentation process changed the red bell pepper samples in the smallest way and the green ones in the highest way.

## 1. Introduction

Fermentation is a process that has been used since ancient times. Once it was discovered and verified that fermented foods had no harmful effects on the human body, fermentation began to be used to preserve food [1]. Lactic fermentation is the enzymatic decomposition of organic substances into simple compounds under anaerobic conditions. This process is carried out by various types of lactic acid bacteria, converting monosaccharides and disaccharides into lactic acid and other compounds with the use of enzymes [2]. There are two types of lactic acid fermentation: spontaneous (fermentation using indigenous microflora) and controlled (fermentation with selected strains of lactic acid bacteria (LAB)) [3,4]. LAB play an important role in this process because they produce lactic acid, which is the main product in fermentation. LAB sugar metabolism is a common feature that connects it to the appropriate microorganisms, but it is also the fact that they are Gram-positive and catalase-negative [1,5,6]. In a domestic setting, the most common fermentation is spontaneous fermentation using autochthonous microflora, i.e., those associated with a particular vegetable. In industrial production, this type of spontaneous fermentation often poses numerous problems in controlling it. For this reason, starter cultures are used [7,8]. The most commonly used *Lactobacillus* cultures for vegetable fermentation are: *Lactiplantibacillus plantarum* (rod-shaped bacteria facultative heterofermentative bacteria classified as thermophiles) [9], *Levilactobacillus brevis* (rod-shaped heterofermentative bacteria) [10], and *Limosilactobacillus fermentum* (rod- or coccoid-shaped, heterofermentative, and a aerotolerant) [11].

Fermented raw materials are mainly dairy products, but it is possible to ferment raw materials such as meat, fish, starch roots, soybeans and other legumes, grains, vegetables, and fruits. At present, more attention has been paid to fermented raw materials of plant origin [12]. Vegetables and fruits are one of the main pillars of the food pyramid. The daily requirement for a group of these products for an adult is 400 g. In developed countries, unfortunately, too little is consumed because consumers are discouraged from greater consumption. This is because vegetables must first be prepared, i.e., washed, peeled, minced, and boiled. Therefore, fermenting vegetables is a very attractive process for their processing. These advantages include a low caloric value, a lower pH in the intestines, and a richness of C and B vitamins. The low-calorie content of silages results from the use of lactic acid fermentation, during which lactic acid bacteria consume sugars and create lactic acid. This lactic acid lowers the intestinal microflora’s pH, which can synthesize enzymes capable of breaking down potentially carcinogenic compounds [13,14]. As much as 75% of the daily requirement of vitamin C needed for the proper functioning of the body is provided by vegetables and fruits, such as currants, strawberries, cabbage, and citrus fruits. In addition, it supports the fight against cancer. A diet rich in β-carotene and vitamin E reduces the number of lipid oxidation processes. This diet helps to inhibit the development of atherosclerosis and also reduces the risk of ischemic disease and heart attack [15,16,17]. Additionally, fermented vegetables retain most of the beneficial properties of the raw material.

Bell pepper (*Capsicum annuum* L.) is a commonly cultivated fruit belonging to the genus *Capsicum* in the *Solanaceae* family, used as a vegetable or spice [16]. Bell peppers can come in several colors (red, green, orange, and yellow), depending mainly on their ability to synthesize chlorophylls or carotenoids and their degree of ripeness. The nutritional value of peppers has been found to directly depend on, among other things, the color of the fruit, the growing conditions, and post-harvest handling [18]. This vegetable makes a significant contribution to the human diet due to, among other things, its antioxidant properties, vitamins (mainly A, C, E), minerals, and numerous polyphenols and pigments. The active compounds of bell peppers can have anti-inflammatory, anti-diabetic, antimicrobial, and immunomodulatory effects. According to the literature and the USDA, peppers are high in water (80–90%) and carbohydrates and low in protein and fat. In addition, peppers contain nutrients, such as vitamins (B, A, D, C, E, and K) and minerals (potassium, sodium, magnesium, calcium, and phosphorus) [19]. Therefore, frequent consumption of peppers provides essential nutrients for human health [20].

Compared to, for example, root vegetables, peppers are characterized by a short storage/shelf life. Due to their lack of stomata, bell peppers require special treatment during storage. Loss of water through the cuticle is the only source of shrinkage, as well as weight and water loss [20,21]. Processing pepper fruits can prolong their storage time. The color of peppers can be associated with important differences in flavor, antioxidant capacity, and content of bioactive compounds. The main carotenoids reported in the literature in red bell peppers are lycopene, capsanthin, cucurbitaxanthin, and zeaxanthin. In yellow bell peppers, the most common are lutein, chlorophyll, zeaxanthin, capsanthin, and carotene. In green peppers, neoxanthin, chlorophyll, lutein, zeaxanthin, and capsanthin have been detected [13,18,19,20,22].

The lactic acid fermentation process of vegetables in the literature includes information on the microflora present in the product, the taste qualities of the product, and partial information on the physical and/or chemical properties of the silage obtained. The information available in the literature indicates a faster process using vegetable-dedicated strains of lactic acid bacteria. Fermentation of vegetables is the most common technique. In this type of lactic acid fermentation, the raw material is penetrated as a result of diffusion into the tissue of the salt, which comes from the brine, and the migration of water with mineral salts and sugars of low molecular weight into the brine [23]. This movement can change the structure and salt-sugar component within the vegetable. Furthermore, the acids, including lactic acid, produced during the fermentation process lower the pH in the brine, which may also involve changes in the pigments sensitive to low pH, such as chlorophylls. 

Therefore, this study aimed to evaluate the effect of the fermentation process with different strains of lactic acid bacteria (LAB) on the chemical and physical properties of three colored bell peppers. *Levilactobacillus brevis* (LB), *Limoslactobacillus fermentum* (LF), and *Lactoplantibacillus plantarum* (LP) were used for fermentation; in addition, spontaneous fermentation with autochthonic bacteria (SF) was carried out. The changes in pigment content, antioxidant potential, and texture were evaluated.

## 2. Results and Discussion

Fermentation of bell peppers was conducted for seven days. In all bell pepper samples, the pH was at level 3.75–3.79, which confirmed that the fermentation process finished. This pH level is recommended for the LAB fermentation of vegetables [1].

### 2.1. Determination of Basic Properties of Fermented Bell Peppers

Three colored bell peppers were characterized by a low dry matter content. The highest dry matter content was seen for red bell peppers, then for yellow, and, lastly, for green. The same relation was observed for the sugar content, but greater differences were observed for the fructose values than the glucose values (Table 1). After the fermentation process, no statistically significant changes in the dry matter values were observed (Table 1), which could be linked to the sugar diffusion into the brine and, at the same time, NaCl diffusion into the tissue. Growth is related to sugar availability. After fermentation, a decrease in glucose and fructose was observed independently of the color of the bell peppers and the type of bacteria (Table 1). In all samples, saccharose was not determined. In red and green bell peppers, bacteria growth used all the glucose from the samples and almost all fructose content. After yellow bell pepper fermentation, the presence of glucose was observed, but not all sugars were used for bacteria growth. The exception was *L*. *Brevis* growth, for which the sugar content did not decrease at the same level as for other fermentation at the same time. However, in the fermentation conditions, growth for all LA bacteria strains was observed and did not differ from the others once. Other authors have stated that *L*. *brevis* prefers pentose sugar over glucose. The growth rates of *L*. *brevis* were found to retain maximum metabolic activity when grown with mixed sugars [24]. The absence of sucrose in the samples of fresh red and yellow bell peppers is not typical. Data for red bell peppers from USDA websites (0.11 g/g of fresh weight) and also from other researchers [25] showed that sucrose is present in red bell peppers. This discrepancy could be related to the type of bell peppers used for our research, which were bell peppers produced in Poland, not in Spain.

### 2.2. Color Coefficients

The color of the peppers was determined for each separately for the top and bottom. Peppers are a plant whose outer layer (top) is covered with a cuticle, which differs significantly in color from the tissue of the inner layer. The brightness (L*) of the top layer for yellow peppers was the highest, while the lowest values were observed for red and green peppers (Table 1). The highest values of the redness coefficient (a*) were observed for red peppers, while the lowest values were observed for green peppers. In contrast, the values of the yellowness coefficient (b*) were highest for yellow peppers, while the values were similar for red and green peppers. Such relationships are related to the dominant main color in each of the analyzed peppers. After the fermentation process, a variation in the values of the color coefficients of the outer layer was observed, which was related to the color of the peppers and, also in some cases, to the type of strain. Only the brightness of the red peppers did not change after the fermentation process, whereas that of the yellow and green peppers did change when fermented with *L*. *Plantarum* and spontaneous fermentation. The redness values after fermentation for the red and yellow peppers decreased, while those for the green peppers increased. The post-fermentation coefficient for yellowness increased for red and green peppers, while no clear behavior was observed for yellow peppers once (Table 1).

The color analysis of the bottom layer showed an increase in the values of redness and yellowness after the fermentation process for each of the analyzed peppers, regardless of the LA strain used. However, the brightness values after the fermentation process depended mainly on the color of the analyzed peppers; a decrease in the brightness values was observed for the red peppers, an increase for the yellow peppers, and no clear trend was observed for the green peppers (Table 1). These color changes can be related to the values observed after fermentation. 

### 2.3. Salt Content

**Figure 1:**
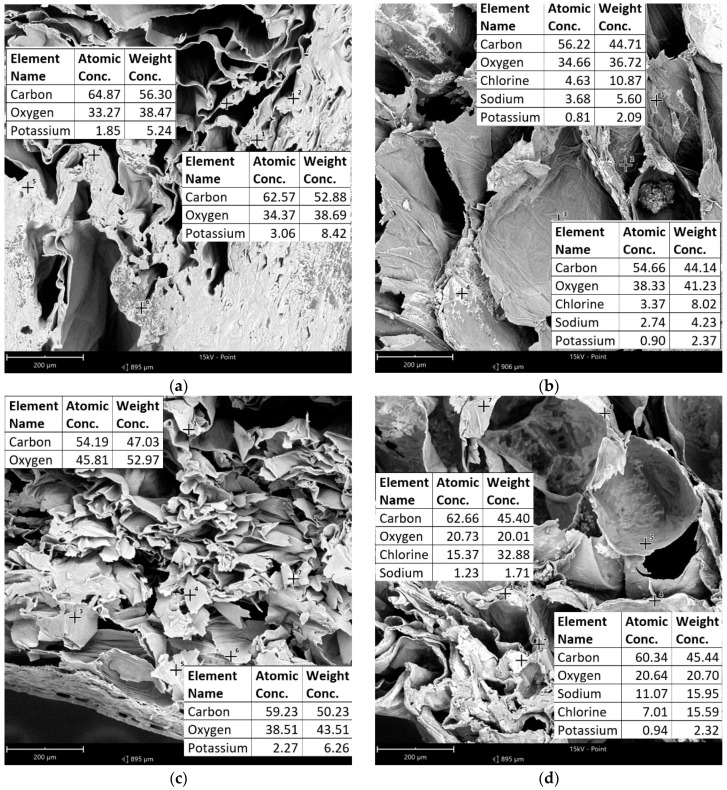

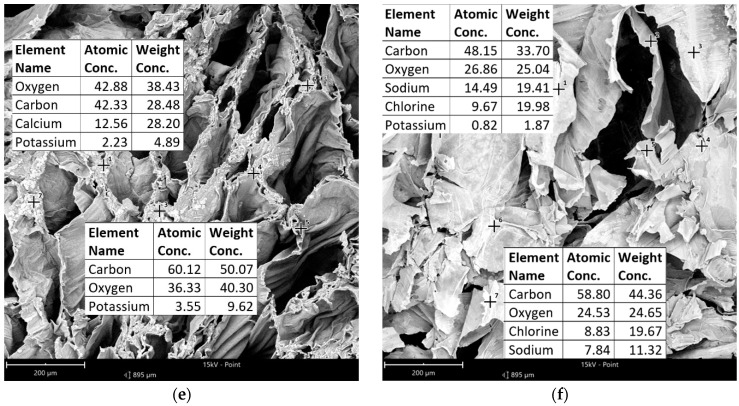
Results of SEM-EDS analysis for bell peppers (**a**) Red_Fresh; (**b**) Red_LP; (**c**) Yellow_Fresh; (**d**) Yellow_LP; (**e**) Green_Fresh; (**f**) Green_LP.

Analysis of SEM-EDS was made in all samples; however, in an article, only pictures of fresh and fermented *L*. *plantarum* were presented (Figure 1). Identification for all samples is placed in the supplementary file in Figure S1. Results obtained from SEM-EDS analysis confirmed salt migration into the tissue (Figure 1). All samples were cut in the middle and then placed in the SEM-EDS for analysis. The results (Figure 1—information in tables, Figure S1 in supplementary file) showed that carbon, oxygen, potassium, and calcium were detected in fresh samples, but no chloride or sodium was observed. In the fermented samples, the detection of Na and Cl was confirmed. 

The main difference in bell peppers was the detection of calcium and nitrogen in green fresh bell pepper, but not in other fresh bell peppers (Figure 1e). From the fermented samples, nitrogen was detected only in green bell peppers fermented spontaneously (Figure S1c). In accordance with USDA data [26] on green bell peppers, a high amount of calcium was present in them in comparison to the red and yellow colors.

### 2.4. Bacteria Count

The pepper was inoculated with *L. fermentum*, *L. brevis*, and *L. plantarum* strains. Additionally, fermentation was carried out with the use of natural microflora found in pepper fruits. These heterofermentative strains (e.g., *L brevis* and *L. fermentum*) produce significant amounts of by-products, such as acetic acid and ethanol, which reduced yields [27]. Other groups of lactic acid bacteria known as facultatively heterofermentative, e.g., *L. plantarum*, use glucose via the Embden–Meyerhof pathway (EMP) to produce lactic acid; they may also have an inducible phosphoketolase (PK) pathway with pentose acting as an inducer. The third group, not tested here, is homofermentative lactic acid bacteria, including, e.g., *Lactobacillus delbrueckii* or *L. lactis,* which cannot convert xylose to lactic acid [28].

Fu and Mathews [29] found that lactic acid fermentation involving *L*. *plantarum* is homogeneous, and it is a homolactic bacterium associated with primary growth. *L*. *brevis* is a heterofermentative strain that has been shown to utilize xylose simultaneously with glucose, which is highly desirable as this strain has no repression of carbon catabolites.

**Figure 2:**
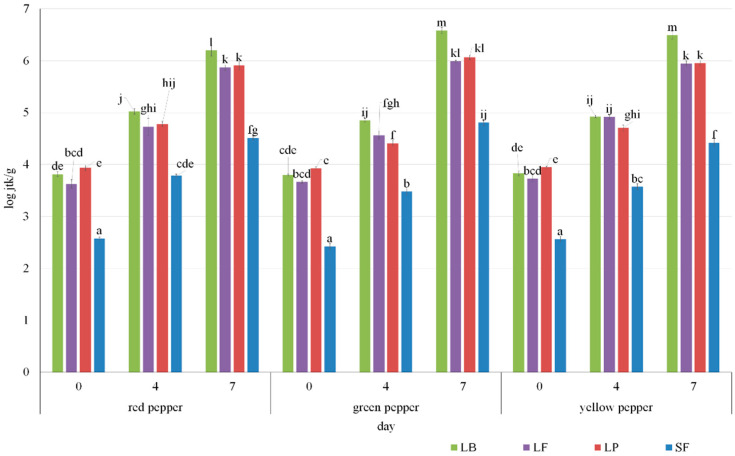
Count of lactic acid bacteria LAB during fermentation of all bell peppers. LB—*Levilactobacillus brevis*; LF—*Limoslactobacillus fermentum*; LP—*Lactoplantibacillus plantarum*; SF—spontaneous fermentation; ^a^, ^b^, ^c^ and specific letters—different indexes for each series mean statistically significant differences for given values at the level of *p* < 0.05.

The number of lactic bacteria increased over the course of fermentation (Figure 2). An increase in the number of LABs by approximately two log cycles was observed during the 7-day fermentation. All types of peppers showed a similar increase in the number of bacteria. No significant influence of the lactic acid bacteria strain used for fermentation on the number of LAB during fermentation was observed.

### 2.5. Texture Analysis

Texture analysis (TPA) measures hardness, elasticity, cohesiveness, and chewiness. Hardness is defined as the force required to break down the sample in the mouth. Springiness is a measure of recovery during chewing; thus, it is a measure of the speed of recovery after the deforming force is removed. The third parameter of cohesiveness is related to the internal bonds that exist in the food; therefore, it informs about the force that keeps the sample cohesive. The last parameter, chewiness, represents the energy required to break down the solid food in order to ingest it [15,30,31]. In the presented research, different colors of fresh bell peppers were similar in all the TPA test results (Table 2). Similar results in springiness and chewiness were observed for fresh green and red bell peppers by Guiné and Barroca [30] and for fresh green peppers by Guiné and Barroca [15]. However, they observed higher results for cohesiveness, which could be related to the product itself or the size of the sample (in our tests, it was a cylindrical shape of sample approximately 5 mm high and with a diameter of 5 mm), while the water content is similar to that presented in our investigations. After the fermentation process, springiness values had significantly increased for all tested bell peppers, which could mean that a longer time of recovery is needed for the samples after fermentation.

For chewiness, an increase in values for yellow and green bell peppers was observed, while for red ones, no statistically significant differences were seen. That means no differences for red pepper will be seen with chewing the fermented bell pepper, while for yellow and green, where the value is almost 3–4 times higher than in fresh ones, this difference will be felt in the mouth. Cohesiveness for all bell peppers was the same as before the process. Only with green bell peppers did higher forces have to be used to bite the piece of pepper after fermentation. In comparison, texture properties for fermented kohlrabi [32] were much higher than in the presented study, which is linked to the structure of the kohlrabi, a root vegetable dissimilar to bell peppers. For three commercial varieties of *Xiaomila* (*Capsicum frutescens* L.), authors [33] observed that the hardness and chewiness of peppers significantly decreased after fermentation, which is different from our experiment. However, those two peppers differed in size, which can influence behavior during the fermentation process. However, the data for the hardness obtained for fresh *Xiaomila* pepper were at a similar level, while the *chewiness was higher* than in our research.

### 2.6. Pigment Amount and Identification

To date, research into the impact of the fermentation process involving LAB has mainly analyzed the effect of bacterial growth and sensory changes on raw materials. According to the data, the growth process of the lactic acid bacteria alone does not have a direct effect on changes or color degradation. However, the resulting metabolic products of bacteria, such as the production of lactic acid, resulted in an environment with a lower pH, which affects the pigments contained in the food. Furthermore, the vegetable fermentation process itself, i.e., flooding of the vegetable with brine of a specific concentration, creates concentration gradients in the system and can lead to an exchange of low-molecular-weight components from the tissue to the brine and from the brine to the raw tissue, which affects (and most of the time increases) the bioavailability of the pigments [34].

**Figure 3:**
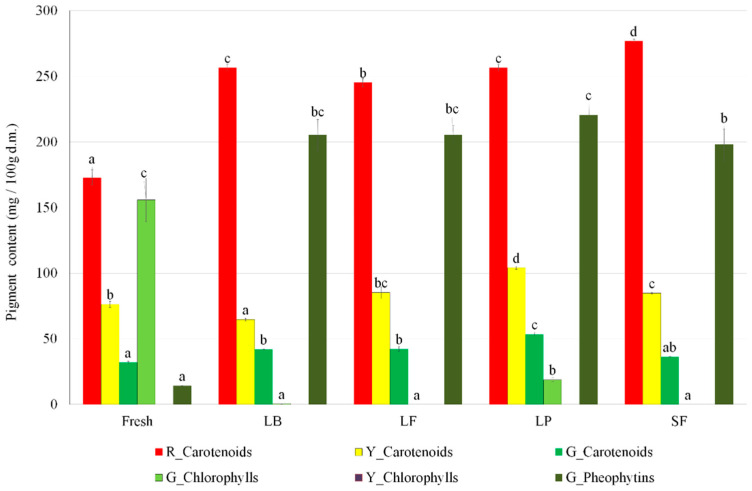
Pigment content; LB—*Levilactobacillus brevis*; LF—*Limoslactobacillus fermentum*; LP—*Lactoplantibacillus plantarum*; SF—spontaneous fermentation; ^a^, ^b^, ^c^ and specific letters—different indexes for series mean statistically significant differences at the level of *p* < 0.05.

Data for pigment identification are placed in Table S1 in the supplementary file. Different colored bell peppers differ in pigment content (Table 3, Figure 3). In red bell pepper, the main part of the pigments is carotenoids-unbounded, monesters, and diesters. Main unbounded carotenoids were detected in increased values: capsorubin, zeaxanthin, lutein, capsanthin, β-cryptoxanthin, and β-carotene. Capsanthin palmitate and capsanthin dipalmitate were detected as the main mono and di-esters of the carotenoids (Table 3). The same monocarotenoids and diesters were detected in yellow bell pepper (fresh and fermented), while in green there was no presence. In yellow and green bell peppers from unbounded carotenoids, only capsorubin, β-cryptoxanthin, and β-carotene were detected. In addition, pheophytin A was detected in yellow bell peppers, while pheophytin B and chlorophylls A and B were detected in green ones (Table 3). Only in green bell peppers did chlorophylls transform into pheophytin after fermentation, which is related to changes in the pH of the brine pH [6]. 

Hallmann, et al. [17] determined carotenoids content in fresh and fermented spontaneous method red bell peppers. Their investigation on fermented red bell peppers showed the presence of β- carotene, cis-βcarotene, α-carotene, capsorubin, cryptoxanthin, cryptoflavin, β-crypotxanthin, α-cryptoxanthin, lutein, zeaxanthin, and cis-ceaxanthin in both organic and conventional bell pepper types tested. Different levels are based on the cultivation, harvest time, and even production system. Analysis of the pigments content divided into the compound was made by Anaya-Esparza, et al. [13]. They compared different results for four colors of bell peppers. They presented the same compounds list as that obtained in our investigations. 

It should be mentioned that chlorophyll is very sensitive and unstable when exposed to light, heat, oxygen, and pH. It can decompose to form pheophytin [35]. Pheophytins are olive-brown pigments and, as the main derivatives of chlorophyll, are formed by the loss of Mg^2+^ from the porphyrin ring under low pH/acid conditions [35,36]. The transformation of chlorophyll to pheophytin changes the color of foods from green to olive-brown, which greatly affects the sensory quality. In addition, according to literature data, lower antioxidant and anti-inflammatory activity are observed after the transformation of chlorophyll to pheophytin [37].

Analysis of the pigment content divided into total carotenoids and pheophytins showed an increase after a fermentation process, while for chlorophylls decrease was observed (Figure 3). These trends were also visible in each chemical compound presented in Table 3. The increase in total carotenoid content in all bell peppers after fermentation is related mostly to the disruption of tissues cells, inside which those carotenoids can be placed. A decrease in chlorophyll and an increase in pheophytins in green bell peppers, as mentioned above, is linked to the chlorophyll-pheophytins transformation in an acidic environment. Analysis of the influence of the starter culture on the pigment content showed that the highest increase in carotenoids was observed in all colors of bell peppers, and pheophytins in green peppers for fermentation with *L*. *Plantarum* as a starter culture. 

### 2.7. Antioxidant Properties

**Figure 4:**
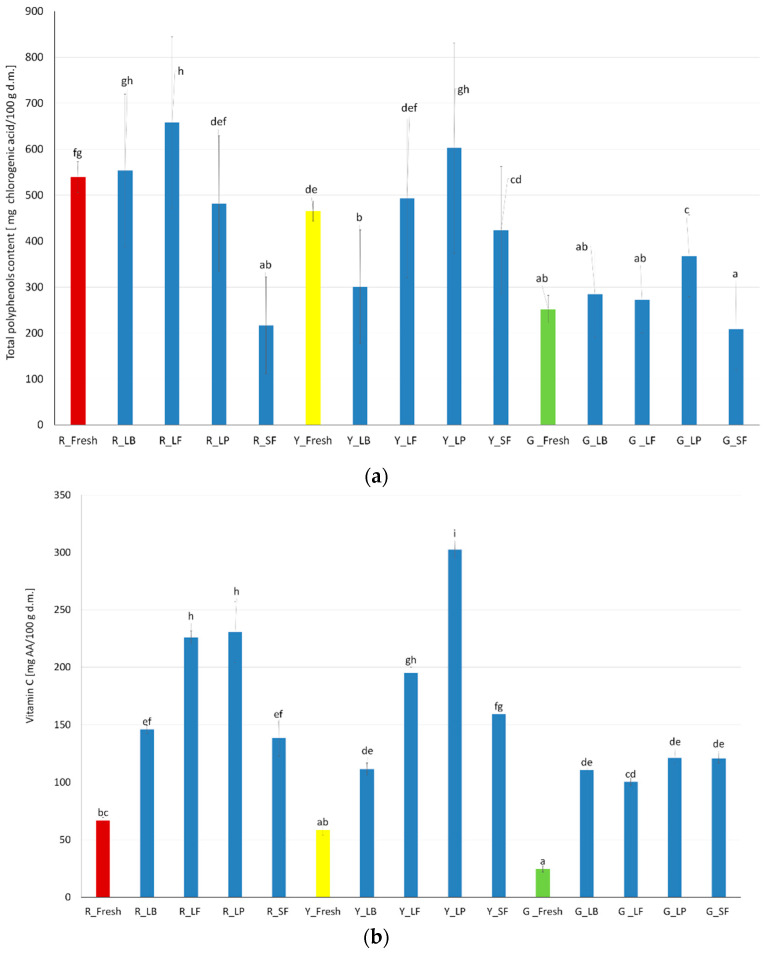

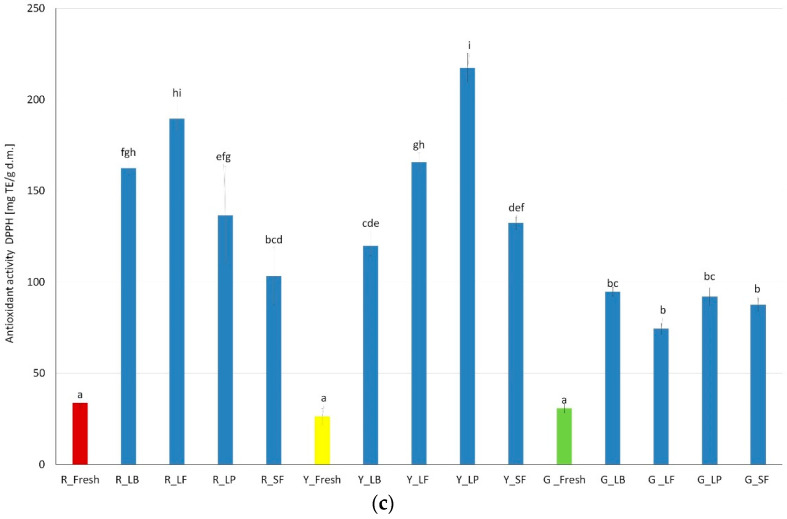
Results of antioxidant tests for bell peppers (**a**) Total polyphenols; (**b**); Vitamin C, (**c**) Antioxidant activity; ^a^, ^b^, ^c^ and specific letters—different indexes on each figure mean statistically significant differences for given values at the level of *p* < 0.05.

Bell peppers are characterized by their different colors and, therefore, different nutritional value. Due to its content of pigments and active compounds, such as phenols and flavonoids, bell peppers have a high antioxidant capacity [13,25,38]. This finding is confirmed by studies on the content (Figure 4a), according to which red bell peppers have the highest amount of phenolic compounds, expressed in mg of chlorogenic acid, followed by yellow and then green, the latter of which is much less. After the fermentation process, no clear trend related to the fluctuation of the phenolic compound was observed. Moreover, there was also no clear effect on their content in relation to the color of the pepper or the strain used. Hallmann et al. [17] tested red bell peppers in polyphenol content. They observed no changes in total phenolic compounds after the fermentation process in two types of red bell peppers. 

In addition to the flavonoid and pigment content, the antioxidant properties may also depend on the vitamin C content. Vitamin C content was tested in fresh and fermented samples. Similar to the phenolic compounds, the highest values were observed in red bell peppers, then in yellow, and the lowest was observed in green bell peppers (Figure 4b). After the fermentation process is conducted independently of the bacteria strains, the vitamin C level rises. In green bell peppers, this increase did not depend on the fermentation bacteria strains, while for red and yellow, higher values were observed for fermentation with *L*. *Plantarum* and *L*. *Fermentum*. Zi, Zhixun, Meiqi, Xuetin, Hongbing, Xiaosong, and Junjie [33] reported the same increase in the vitamin C content in chili pepper after fermentation in comparison to the fresh pepper. However, in their research, the vitamin C in the red and green peppers was much higher than in ours. The authors stated that this level of vitamin C is related to longer sunlight when harvest is during summer.

The antioxidant values are presented in Figure 4c. They showed a similar correlation as was seen for vitamin C: the highest values are observed for red and yellow bell peppers fermented with *L*. *plantarum*.

## 3. Materials and Methods

### 3.1. Materials 

Bell pepper (*Capsicum annuum* L.)—red (R), yellow (Y), and green (G)—were purchased from the Bronisze market (Warsaw, Poland) and stored in a temperature range of 4 to 6 °C. As an inoculum for fermentation, three strains were selected: *Levilactobacillus brevis* KKP 804 (LB), *Limosilactobacillus fermentum* KKP 811 (LF), and *Lactoplantibacillus plantarum* ATCC 4080 (LP). Spontaneous fermentation (SF) with autochthonic microbiota took place, as well. Reference strains were obtained from the Collection of Industrial Microorganisms (KKP, Warsaw, Poland) and American Type Culture Collection (ATCC, Manassas, VA, USA). Bacterial inoculum was prepared in physiological saline (0.85% NaCl) at a concentration of ~1 × 10^7^ CFU/mL [39].

### 3.2. Technological Treatment 

#### Fermentation Process 

Fermentation was carried out in the cut vegetables. It was washed before pickling. In addition, the peppers that were to be inoculated with dedicated lactic acid bacteria were subjected to a 3 min soak in sodium hypochlorite to remove autochthonous microflora. Afterward, rectangles of about 2 × 3 cm were cut from the bell peppers. The cut vegetables (about 250 g) were placed into 500 mL jars, to which 230 mL of 2% *v*/*v* of NaCl solution was added. Then inoculum in the amount of 1% of the water volume was added. For spontaneous fermentation (SF), no inoculum was added. Jars were closed and kept in an incubator at a stable temperature of 28 °C. Fermentation was carried out for 7 days. All experiments were performed in duplicate. 

### 3.3. Analytical Method 

#### 3.3.1. Dry Matter Content 

The dry matter was determined by the gravimetric method. Approximately 0.6–1 g of sample was placed in a dish and dried by the vacuum drying method (Memmert VO400, Schwabach, Germany) under the pressure of 10 mPa at 75 °C for 24 h until constant weight. Measurements were made in triplicate. 

#### 3.3.2. Color Parameters 

Analysis of color was made in CR-5 (Konica Minolta Sensing Inc., Osaka, Japan) in a CIE L*a*b* system. Illuminant D65, angle 2°, and calibration with white and black color. For all bell peppers, separate colors for top layer and bottom were taken. It is related to the huge difference in bell peppers. All measurements were made in 10 repetitions [40].

#### 3.3.3. Texture Analysis

To determine the textural properties of raw and fermented vegetable samples, texture profile analysis were carried out. Texture profile analysis (TPA) of all the samples was performed using a Texture Analyzer TA.XT2i instrument (Stable Micro Systems, Surrey, UK) [15]. The cylindrical shape samples approximately 5 mm high and with a diameter of 5 mm were cut. Texture profile analysis was carried out by 2 compression cycles using a 75 mm diameter plunger, with a 5 s period of time between cycles. In addition, 5 kg force load cell and 0.5 mm/s test speed were used for the test. The textural properties of springiness, cohesiveness, and chewiness were calculated. Measurements were made in ten repetitions.

#### 3.3.4. Vitamin C

The vitamin C content was determined using ultra-performance liquid chromatography (UPLC) according to Spínola et al. [41] with modification. The material was crushed in the analytical mill and extracted with a mixture of 3% MPA—8% acetic acid—1 mM EDTA. After centrifugation, the supernatant was filtered through 0.2 μm GHP ASCORDISC filters (Milipore, Burlington, MA, USA). An Acquity UPLC system (Waters Corporation, Milford, MA, USA) with a Waters Acquity UPLC photodiode array (PDA) detection system and EmpowerTM software (Waters Corporation, Milford, MA, USA) was used to record the detection signal and process the peak areas. The chromatograph is equipped with an Acquity HSS T3 analytical column (100 × 2.1 mm, 1.8 μm particle size). An isocratic mobile phase consisting of aqueous 0.1% (*v*/*v*) formic acid with a specific flow rate was used. The detection wavelength for the PDA was 245 nm. The L-ascorbic acid in the samples was determined by comparing it with the retention time of the standard and matching it with the UV absorption spectrum. Measurements were made in two repetitions.

#### 3.3.5. Sugar Content

Sugar content was analyzed by HPLC (High Performance Liquid Chromatography) with refractive index detection (Waters, Milford, CT, USA) [42]. The material was homogenized in a laboratory mill and extracted with ultra-pure water (MilliQ redistilled water, 80 °C), then centrifugation for 5 min at 6000 rpm take place. The supernatant was filtered using a 0.22 μm GHP syringe filter and injected into the system. For separation, a Sugar-Pak I column with precolumn (Waters, Milford, CT, USA) was used. The temperature of the column thermostat was 90 °C and the detector temperature was 60 °C. Distilled water was used as the flow rate for the mobile phase with a flow rate of 0.6 mL/ min. The sugar content was determined on the basis of calibration curves obtained separately for each of the standards analyzed: sucrose, glucose, and fructose. Measurements were made in two repetitions.

#### 3.3.6. SEM-EDS Analysis

Mineral composition was determined by scanning electron microscopy—energy dispersive X-ray spectroscopy (SEM–EDS) using an energy dispersion spectrometer (SDD type) integrated with a scanning electron microscope (Phenom-World B.V., FEI Company, Eindhoven, The Netherlands). Before analysis, the freeze-drying process was used (48 h, drying process temperatures 25 °C). The dried bell pepper samples were cut in half and placed on double-sticky tape. The analysis was carried out on at least four randomly selected points.

#### 3.3.7. Pigment Content

The sample size of approximately 2 g was extracted using three portions of 10 mL of acetone/hexane 1:1 (*v*/*v*) containing butylated hydroxytoluene (0.5 g/L). The organic phases were collected and washed with sodium chloride solution (50 g/L) to remove water residues. Two milliliters were evaporated in vacuo and the residue was dissolved in isopropanol, membrane filtered (0.2 µm), and used for analysis.

The UPLC-HRMS analysis was conducted on a Vanquish LC system connected to a Q Exactive Focus Orbitrap mass spectrometer (both Thermo Fischer Scientific, Waltham, MA, USA) using their own modification of the method presented by Schweiggert et al. [43]. Samples were separated on a Cortex UPLC C18 column, 1.6 µm, 2.1 × 100 mm (Waters, Milford, MA, USA), and kept at a temperature of 25 °C, which was eluted at a flow rate of 0.4 mL/min using a gradient consisting of methanol/MTBE/water 81:15:4 (*v*/*v*/*v*, solvent A) and methanol/MTBE/water 81:15:4 (*v*/*v*/*v*, solvent B) as follows: 0 min 0% B, 22 min 30% B, 32 min 51% B, 55 min 63% B, 60 min 100% B, 65 min 100% B, 70 min 0% B. The injection volume was 5 µL.

The mass spectrometer was fitted with an atmospheric pressure chemical ionization (APCI) source. The data acquisition and processing were performed using XCalibur software. The carotenoids and their esters were identified using the data from the original paper of Schweiggert et al. [43], while chlorophylls and pheophytins used the data of Huang et al. [44]. Positive mass spectra were recorded in the m/z range of 200–1400. The capillary temperature was set to 450 °C, while the spray voltage was 3 kV. Nitrogen was used as both sheath gas and auxiliary gas, with flow rates of 20.0 L/min and 5.0 L/min, respectively. The entire ion fragmentation approach (AIF) was used for the identification of analytes, while standard chlorophyll a, β-carotene, and zeaxanthin were used for their quantification. Measurements were made in two repetitions.

#### 3.3.8. Total Phenolic Content and Antioxidant Activity

Antioxidant activity with Folin reagent was determined by using a spectrophotometric method. The extract was mixed with distilled water and a Folin-Ciocalteu solution, and after 3 min supersaturated sodium carbonate was added. After 1 h of incubation at 25 °C, the absorbance of the solution was measured at 750 nm [42]. A calibration curve standard for chlorogenic acid was established. Measurements were made in two repetitions.

In order to generate free radicals, a stock solution of DPPH was prepared within 24 h before the analysis [45]. In the beginning, in a 100 mL volumetric flask, a 25 mg of 2,2-diphenyl-1-picrylhydrazyl was weighed and made with a 99% methanol solution up to 100 mL. Working solutions of the radicals were prepared by diluting the stock solutions with 80% ethanol to obtain a concentration showing absorption in a 1 cm cuvette at a wavelength of 515 nm for DPPH at about 0.680–0.720 AU (absorbance unit). Reactions were performed in 96-well plates. At the same time, the absorbance of the radical working solutions was checked. Measurements were made in two repetitions. The antiradical activity was determined from the decrease in the absorbance of the radical solution in the presence of an antioxidant and expressed as mg Trolox Equivalent/g material.

#### 3.3.9. Determination of the Number of Lactic Acid Bacteria 

Total count by pour plate method was used to enumerate viable cells. Pepper samples were serially diluted using sterile saline (0.85% NaCl, Biomaxima, Poland). The samples were streaked onto plates de Man Rogosa and Sharpe Agar (MRS, Biomaxima, Poland) agar and incubated at 28 °C ± 1 °C for 48 h ± 4 h. The number of colonies grown was counted (ProtoCOL 3—Automatic colony counting and zone measuring, Synbiosis, USA) and recorded as log CFU per g. The samples were analyzed in triplicates. The analysis was performed after inoculation (in the case of samples with the addition of lactic acid bacteria) and on the day of starting spontaneous fermentation, and then on the 4th and 7th day of fermentation.

### 3.4. Statistical Treatment

The results obtained were subjected to a statistical analysis using Statistica 13 software (StatSoft, Warsaw, Poland). An analysis of multiple variances (ANOVA) was performed to assess the significance of the physical and chemical properties of the peppers. Results were divided into homogeneous groups using the Tukey HSD method (α = 0.05). Letters from homogeneous groups. The other parameters were determined using MS Excel 2019. 

## 4. Conclusions

A similar increase was observed for the starter cultures in all samples tested. After the fermentation process, significant conversion of chlorophylls to pheophytins, as well as increased vitamin C and total polyphenols were observed, which affected the antioxidant activity of the samples. Texture properties showed no differences in hardness, while higher elasticity was observed, indicating that a long time would be needed for re-deformation of the fermented samples compared to the fresh ones. No differences in chewing values were observed for the red peppers, which may mean that the consumer will not experience differences between fresh and fermented peppers when biting and chewing; however, other (not tested, but observed) aroma and flavor characteristics will significantly differentiate these samples. It can be concluded that the fermentation process altered the red pepper samples the least and the green pepper samples the most. 

## Figures and Tables

**Table 1:** Results of selected physical and chemical properties of fresh and fermented bell peppers.

Sample Name	Dry Matter (%)	Color Parameters Top			Color Parameters Bottom			Glucose (g/100 g d.m.)	Fructose (g/100 g d.m.)	Total Sugars (g/100 g d.m.)
		L*	a*	b*	L*	a*	b*			
R_Fresh	6.97 ± 0.14 ^b^	29.8 ± 0.9 ^ab^	34.2.4 ^h^	17.8 ± 1.0 ^a^	35.9 4.7 ^d–f^	23.2 ± 1.9 ^e^	16.9 ± 3.7 ^a^	22.8 ± 1.3 ^e^	19.8 ± 0.9 ^c^	42.5 ± 0.5 ^g^
R_LB	4.57 ± 0.03 ^ab^	32.2 ± 0.5 ^ab^	29.3 ± 0.3 ^g^	32.9 ± 5.7 ^de^	31.7 ± 1.4 ^bc^	33.88 ± 3.93 ^f^	25.10 ± 3.98 ^bc^	24.6 ± 2.9 ^e^	12. ± 1.1 ^b^	36.6 ± 1.8 ^e^
R_LF	4.71 ± 0.36 ^ab^	34.5 ± 5.0 ^b^	23.7 ± 4.8 ^f^	31.1 ± 5.2 ^cd^	33.9 ± 1.1 ^a–c^	35.90 ± 1.98 ^g^	27.38 ± 0.73 ^cd^	0.00 ± 0.^a^	0.2 ± 0.0 ^a^	0.2 ± 0.0 ^a^
R_LP	4.60 ± 0.03 ^ab^	29.0 ± 4.0 ^a^	22.4 ± 3.8 ^f^	25.7 ±3.7 ^b^	30.3 ± 3.3 ^b^	35.08 ± 3.64 ^g^	31.07 ± 0.33 ^c–f^	0.0 ± 0.0 ^a^	0.2 ± 0.0 ^a^	0.2 ± 0.0 ^a^
R_SF	3.37 ± 0.26 ^ab^	29.2 ± 2.4 ^a^	19.6 ± 1.5 ^e^	21. ± 1.6 ^a^	25.5 ± 0.5 ^a^	24.35 ± 0.50 ^e^	26.85 ± 5.08 ^cd^	0.0 ± 0.0 ^a^	0.2 ± 0.0 ^a^	0.2 ± 0.0 ^a^
Y_Fresh	5.39 ± 0.45 ^ab^	49.6 ± 3.1 ^ef^	0.7 ± 0.3 ^d^	43.9 ± 5.1 ^g^	42.4 ± 1.7 ^gh^	−0.2 ± 0.7 ^bc^	25.8 ± 1.3 ^b–d^	21.8 ± 1.4 ^e^	17.8 ± 0.5 ^c^	39.50 ± 0.9 ^f^
Y_LB	5.97 ± 0.39 ^ab^	51.2 ± 4.4 ^f^	−2.2 ± 0.6 ^bc^	43.1 ± 6.2 ^g^	49.0 ± 4.4 ^j^	2.2 ± 2.5 ^d^	68.0 ± 10.3 ^i^	17.8 ± 0.6 ^d^	11.3 ± 1.3 ^b^	29.8 ± 0.8 ^d^
Y_LF	4.75 ± 0.22 ^ab^	44.8 ± 1.0 ^de^	−2.622 ± 0.2 ^bc^	39.5 ± 0.7 ^fg^	48.4 ± 2.1 ^ij^	−0.5 ± 0.6 ^bc^	58.9 ± 1.7 ^h^	1.9 ± 0.2 ^ab^	0.5 ± 0.1 ^a^	2.4 ± 0.3 ^ab^
Y_LP	3.78 ± 1.47 ^ab^	34.4 ± 6.9 ^b^	−0.712 ± 1.2 ^cd^	36.5 ± 1.9 ^ef^	46.2 ± 3.7 ^i^	1.9 ± 0.2 ^d^	52.6 ± 11.5 ^g^	3.9 ± 0.2 ^b^	0.8 ± 0.1 ^a^	4.6 ± 0.3 ^b^
Y_SF	4.20 ± 0.40 ^ab^	42.4 ± 4.7^8 d^	−0.9 ± 0.2 ^cd^	51.3 ± 5.3 ^h^	43.3 ± 0.3^4^	1.4 ± 1.1 ^cd^	48.1 ± 1.3 ^g^	1.4 ± 0.2 ^ab^	0.6 ± 0.1 ^a^	1.9 ± 0.2 ^ab^
G_Fresh	4.31 ± 0.35 ^ab^	33.4 ± 0.9 ^ab^	−12.5 ± 0.5 ^a^	19.8 ± 1.2 ^a^	39.3 ± 1.8 ^fg^	−9.9 ± 0.5 ^a^	17.9 ± 1.2 ^a^	9.9 ± 0.3 ^c^	13.9 ± 1.8 ^b^	23.8 ± 1.4 ^c^
G_LB	3.62 ± 0.18 ^ab^	35.4 ± 2.0 ^b^	−1.9 ± 0.1 ^bc^	29.1 ± 0.6 ^bc^	39.0 ± 1.3 ^fg^	−1.9 ± 0.2 ^b^	29.1 ± 2.3 ^c–e^	3.7 ± 0.1 ^b^	0.3 ± 0.0 ^a^	4.1 ± 0.1 ^b^
G_LF	3.52 ± 0.25 ^ab^	33.3 ± 1.9 ^b^	−1.1 ± 0.6 ^cd^	28.2 ± 0.2 ^bc^	34.4 ± 2.2 ^bc^	2.1 ± 0.4 ^d^	34.4 ± 5.4 ^ef^	0.0 ± 0.0 ^a^	0.2 ± 0.0 ^a^	0.2 ± 0.0 ^a^
G_LP	2.82 ± 0.20 ^a^	43.7 ± 6.7 ^d^	−3.5 ± 0.8 ^b^	25.7 ± 0.4 ^b^	30.8 ± 2.5 ^b^	1.5 ± 0.2 ^cd^	29.6 ± 7.3 ^a–c^	0.0 ± 0.0 ^a^	0.0 ± 0.0 ^a^	0.0 ± 0.0 ^a^
G_SF	3.54 ± 0.01 ^ab^	31.0 ± 2.7 ^ab^	−1.1 ± 0.3 ^cd^	26.0 ± 0.4 ^b^	37.4 ± 1.4 ^ef^	1.0 ± 0.5 ^b–d^	36.1 ± 1.8 ^f^	0.0 ± 0.0 ^a^	0.4 ± 0.0 ^a^	0.4 ± 0.0 ^a^

R,Y,G—Red, Yellow, or Green bell peppers; LB—*Levilactobacillus brevis*; LF—*Limoslactobacillus fermentum*; LP—*Lactoplantibacillus plantarum*; SF—spontaneous fermentation; L*—lightness; a*—redness/greenies; b*—yellowness/blueness; ^a^, ^b^, ^c^ and specific letters—different indexes for each column mean statistically significant differences for given values at the level of *p* < 0.05.

**Table 2:** Texture analysis results.

Sample Name	Hardness [N]	Springiness [%]	Cohesiveness [-]	Chewiness [N]
R_Fresh	34.07 ± 10.97 ^a–c^	38.94 ± 11.90 ^ab^	0.33 ± 0.14 ^a^	3.88 ± 1.87 ^a–d^
R_LB	36.12 ± 9.87 ^bc^	64.11 ± 14.70 ^de^	0.19 ± 0.04 ^a^	4.46 ± 2.37 ^cd^
R_LF	32.89 ± 10.11 ^ab^	69.14 ± 10.18 ^de^	0.24 ± 0.11 ^a^	5.44 ± 1.74 ^cd^
R_LP	35.88 ± 14.03 ^a–c^	75.58 ± 14.54 ^e^	0.24 ± 0.13 ^a^	4.66 ± 2.16 ^cd^
R_SF	27.73 ± 14.34 ^a–c^	65.99 ± 16.00 ^b–e^	0.20 ± 0.03 ^a^	4.30 ± 1.75 ^a–d^
Y_Fresh	22.90 ± 4.74 ^a^	37.28 ± 19.87 ^a–c^	0.14 ± 0.05 ^a^	1.16 ± 0.78 ^a^
Y_LB	29.55 ± 6.48 ^a^	54.81 ± 13.65 ^b–d^	0.18 ± 0.04 ^a^	3.54 ± 1.67 ^a–c^
Y_LF	36.66 ± 6.66 ^a–c^	62.39 ± 14.08 ^b–e^	0.25 ± 0.12 ^a^	5.46 ± 1.89 ^b–d^
Y_LP	33.32 ± 8.88 ^ab^	62.92 ± 12.97 ^c–e^	0.26 ± 0.08 ^a^	6.40 ± 1.98 ^d^
Y_SF	31.04 ± 11.42 ^a–c^	60.00 ± 17.65 ^b–e^	0.21 ± 0.10 ^a^	4.30 ± 2.48 ^cd^
G_Fresh	32.23 ± 3.60 ^ab^	21.30 ± 11.77 ^a^	0.18 ± 0.03 ^a^	1.50 ± 0.87 ^ab^
G_LB	50.47 ± 14.39 ^c^	57.03 ± 17.04 ^b–e^	0.18 ± 0.03 ^a^	4.44 ± 1.68 ^b–d^
G_LF	44.48 ± 13.00 ^bc^	67.15 ± 15.00 ^de^	0.31 ± 0.16 ^a^	6.38 ± 2.69 ^d^
G_LP	44.42 ± 12.28 ^bc^	67.12 ± 18.01 ^de^	0.18 ± 0.06 ^a^	4.78 ± 1.57 ^cd^
G_SF	36.43 ± 15.00 ^a–c^	66.70 ± 10.99 ^b–e^	0.22 ± 0.06 ^a^	5.52 ± 1.70 ^b–d^

R, Y, G—Red, Yellow or Green bell peppers; LB—*Levilactobacillus brevis*; LF—*Limoslactobacillus fermentum*; LP—*Lactoplantibacillus plantarum*; SF—spontaneous fermentation; ^a^, ^b^, ^c^ and specific letters—different indexes for each column mean statistically significant differences for given values at the level of *p* < 0.05.

**Table 3:** Results of chlorophylls and carotenoids pigments in samples (mg/100 g d.m.; described as average ± standard deviation).

Sample Name	Chlorophylls and Derivatives				Unbounded Carotenoids						Carotenoid Monoesters						Carotenoid Diesters									
	Chlorophyll A	Chlorophyll B	Pheophytin A	Pheophytin B	β-carotene	Capsanthin	Capsorubin	Zeaxanthin	Lutein	β-cryptoxanthin	cap-lau	cap-myr	cap-pal	zea-lau	zea-myr	zea-pal	cap-lau-lau	cap-lau-myr	cap-myr-myr	cap-lau-pal	cap-myr-pal	cap-pal-pal	zea-myr-myr	zea-lau-pal	zea-myr-pal	zea-pal-pal
R_Fresh	0	0	0	0	7.92 ± 0.22 ^f^	9.49 ± 0.43 ^a^	50.44 ± 1.97 ^c^	45.33 ± 1.07 ^a^	19.64 ± 0.96 ^a^	9.40 ± 0.31 ^a^	0.58 ± 0.03 ^a^	2.67 ± 0.05 ^a^	13.98 ± 0.27 ^b^	0.34 ± 0.04 ^a^	0.66 ± 0.04 ^a^	2.09 ± 0.07 ^a^	0.10 ± 0.02 ^a^	0.42 ± 0.03 ^a^	0.50 ± 0.02 ^a^	0.24 ± 0.00 ^a^	0.98 ± 0.05 ^a^	5.74 ± 0.02 ^b^	0.30 ± 0.02 ^a^	0.23 ± 0.00 ^a^	0.48 ± 0.05 ^a^	1.45 ± 0.02 ^a^
R_LB	0	0	0	0	12.39 ± 0.14 ^h^	12.72 ± 0.05 ^c^	77.42 ± 0.88 ^fg^	67.26 ± 0.99 ^b^	28.26 ± 0.14 ^b^	13.48 ± 0.45 ^c^	0.73 ± 0.02 ^b^	3.89 ± 0.06 ^b^	20.89 ± 0.14 ^d^	0.49 ± 0.05 ^b^	0.91 ± 0.14 ^b^	3.07 ± 0.05 ^c^	0.16 ± 0.02 ^b^	0.54 ± 0.08 ^b^	0.63 ± 0.03 ^bc^	0.31 ± 0.06 ^a–c^	1.45 ± 0.08 ^c^	8.34 ± 0.26 ^d^	0.42 ± 0.03 ^b^	0.36 ± 0.02 ^c^	0.68 ± 0.09 ^bc^	2.22 ± 0.08 ^bc^
R_LF	0	0	0	0	11.09 ± 0.47 ^g^	12.07 ± 0.08 ^b^	71.20 ± 1.10 ^ef^	67.01 ± 1.32 ^b^	27.43 ± 0.15 ^b^	13.34 ± 0.20 ^c^	0.76 ± 0.06 ^b^	3.63 ± 0.12 ^b^	20.33 ± 0.44 ^d^	0.46 ± 0.08 ^b^	0.93 ± 0.06 ^b^	2.97 ± 0.03 ^b^	0.15 ± 0.00 ^b^	0.49 ± 0.03 ^ab^	0.61 ± 0.02 ^b^	0.35 ± 0.02 ^c^	1.40 ± 0.06 ^c^	7.45 ± 0.21 ^c^	0.41 ± 0.02 ^b^	0.32 ± 0.03 ^bc^	0.63 ± 0.11 ^ab^	2.12 ± 0.06 ^cd^
R_LP	0	0	0	0	12.35 ± 0.58 ^h^	12.79 ± 0.3 ^c^	75.78 ± 0.43 ^fg^	67.85 ± 0.74 ^b^	28.68 ± 0.63 ^b^	13.96 ± 0.28 ^c^	0.78 ± 0.03 ^b^	3.90 ± 0.08 ^b^	20.58 ± 0.11 ^d^	0.48 ± 0.00 ^b^	0.95 ± 0.02 ^b^	3.16 ± 0.05 ^c^	0.14 ± 0.02 ^b^	0.58 ± 0.02 ^b^	0.70 ± 0.03 ^c^	0.32 ± 0.02 ^bc^	1.46 ± 0.00 ^c^	8.42 ± 0.02 ^d^	0.39 ± 0.03 ^b^	0.32 ± 0.02 ^bc^	0.71 ± 0.02 ^c^	2.28 ± 0.03 ^d^
R_SF	0	0	0	0	12.41 ± 0.06 ^h^	12.62 ± 0.31 ^bc^	85.68 ± 3.19 ^h^	76.80 ± 3.50 ^c^	32.32 ± 0.42 ^c^	17.36 ± 0.06 ^h^	0.53 ± 0.04 ^a^	2.83 ± 0.15 ^a^	17.78 ± 0.49 ^c^	0.46 ± 0.06 ^b^	0.90 ± 0.02 ^b^	2.85 ± 0.17 ^b^	0.15 ± 0.00 ^b^	0.53 ± 0.04 ^b^	0.68 ± 0.04 ^c^	0.25 ± 0.02 ^ab^	1.25 ± 0.04 ^b^	8.33 ± 0.29 ^d^	0.36 ± 0.04 ^ab^	0.27 ± 0.04 ^ab^	0.53 ± 0.04 ^ab^	1.97 ± 0.10 ^b^
Y_Fresh	0	0	0.37 ± 0.08 ^a^	0	2.19 ± 0.24 ^c–e^	0	58.57 ± 2.18 ^d^	0	0	15.01 ± 0.05 ^ef^	0	0	0.20 ± 0.03 ^a^	0	0	0	0	0	0	0	0	0.35 ± 0.03 ^a^	0	0	0	0
Y_LB	0	0	0.31 0.04 ^a^	0	1.92 ± 0.06 ^a–e^	0	49.07 ± 0.82 ^c^	0	0	13.47 ± 0.14 ^c^	0	0	0.14 ± 0.01 ^a^	0	0	0	0	0	0	0	0	0.25 ± 0.02 ^a^	0	0	0	0
Y_LF	0	0	0.40 0.02 ^a^	0	2.41 ± 0.09 ^de^	0	65.30 ± 4.30 ^de^	0	0	17.09 ± 0.06 ^h^	0	0	0.20 ± 0.00 ^a^	0	0	0	0	0	0	0	0	0.37 ± 0.00 ^a^	0	0	0	0
Y_LF	0	0	0.48 0.04 ^a^	0	3.01 ± 0.19 ^e^	0	79.44 ± 0.65 ^g^	0	0	21.05 ± 0.22 ^j^	0	0	0.25 ± 0.02 ^a^	0	0	0	0	0	0	0	0	0.41 ± 0.02 ^a^	0	0	0	0
Y_SF	0	0	0.43 0.07 ^a^	0	2.27 ± 0.05 ^c–e^	0	64.20 ± 0.57 ^de^	0	0	18.02 ± 0.10 ^i^	0	0	0.18 ± 0.02 ^a^	0	0	0	0	0	0	0	0	0.20 ± 0.02 ^a^	0	0	0	0
G_Fresh	139.20 ± 14.79 ^c^	16.66 ± 1.52 ^b^	9.97 ± 0.51 ^a^	4.43 ± 0.70 ^a^	1.24 ± 0.06 ^ab^	0	18.84 ± 0.55 ^a^	0	0	11.98 ± 0.28 ^b^	0	0	0	0	0	0	0	0	0	0	0	0	0	0	0	0
G_LB	0.57 ± 0.10 ^a^	0	182.85 ± 11.44 b^c^	22.61 ± 0.16 ^b^	1.41 ± 0.04 ^ab^	0	25.31 ± 0.18 ^ab^	0	0	15.43 ± 0.12 ^fg^	0	0	0	0	0	0	0	0	0	0	0	0	0	0	0	0
G_LF	0	0	183.47 ± 6.27 ^bc^	22.07 ± 0.50 ^b^	1.46 ± 0.10 ^a–c^	0	24.65 ± 2.13 ^ab^	0	0	16.16 ± 0.06 ^g^	0	0	0	0	0	0	0	0	0	0	0	0	0	0	0	0
G_LP	17.31 ± 1.23 ^b^	1.54 ± 0.18 ^a^	193.90 ± 2.26 ^c^	26.78 ± 0.58 ^c^	2.01 ± 0.1 ^b–d^	0	31.10 ± 1.05 ^b^	0	0	20.54 ± 0.08 ^j^	0	0	0	0	0	0	0	0	0	0	0	0	0	0	0	0
G_SF	0	0	176.20 ± 10.84 ^b^	21.75 ± 1.18 ^b^	1.03 ± 0.06 ^a^	0	21.13 0.18 ^a^	0	0	14.30 ± 0.10 ^de^	0	0	0	0	0	0	0	0	0	0	0	0	0	0	0	0

R,Y,G—Red, Yellow or Green bell peppers; LB—*Levilactobacillus brevis*; LF—*Limoslactobacillus fermentum*; LP—*Lactoplantibacillus plantarum*; SF—spontaneous fermentation; Cap—capsanthin; lau—laurate; myr—myristate; pal- palmitate; ^a^, ^b^, ^c^ and specific letters—different indexes for each column mean statistically significant differences for given values at the level of *p* < 0.05.

## Data Availability

Data available from the corresponding author.

## Supplementary Materials

The following supporting information can be downloaded at: https://www.mdpi.com/article/10.3390/molecules27238637/s1, Figure S1. Results of SEM-EDS analysis for bell peppers (a) Red; (b) Yellow; (c) Green. Table S1. Compound identification parameters.

## References

[1] Niakousari M., Razmjooei M., Nejadmansouri M., Barba F.J., Marszałek K., Koubaa M., Koubaa M., Barba F.J., Roohinejad S. (2021). Current Developments in Industrial Fermentation Processes. Fermentation Processes: Emerging and Conventional Technologies.

[2] Jackson R.S., Jackson R.S. (2020). Chapter 7—Fermentation. Wine Science.

[3] Mustafa S.M., Chua L.S., Grumezescu A.M., Holban A.M. (2020). 13—Green Technological Fermentation for Probioticated Beverages for Health Enhancement. Biotechnological Progress and Beverage Consumption.

[4] Di Cagno R., Coda R., De Angelis M., Gobbetti M. (2013). Exploitation of vegetables and fruits through lactic acid fermentation. Food Microbiol..

[5] Khubber S., Marti-Quijal F.J., Tomasevic I., Remize F., Barba F.J. (2022). Lactic acid fermentation as a useful strategy to recover antimicrobial and antioxidant compounds from food and by-products. Curr. Opin. Food Sci..

[6] Marszałek K., Woźniak Ł., Wiktor A., Szczepańska J., Skąpska S., Witrowa-Rajchert D., Saraiva J.A., Lorenzo J.M., Barba F.J., Koubaa M., Barba F.J., Roohinejad S. (2021). Emerging Technologies and Their Mechanism of Action on Fermentation. Fermentation Processes: Emerging and Conventional Technologies.

[7] Garcia C., Remize F. (2022). Lactic acid fermentation of fruit and vegetable juices and smoothies: Innovation and health aspects. Lactic Acid Bacteria in Food Biotechnology.

[8] Kiczorowski P., Kiczorowska B., Samolińska W., Szmigielski M., Winiarska-Mieczan A. (2022). Effect of fermentation of chosen vegetables on the nutrient, mineral, and biocomponent profile in human and animal nutrition. Sci. Rep..

[9] Li Y., Ten M.M.Z., Zwe Y.H., Li D. (2022). *Lactiplantibacillus plantarum* 299v as starter culture suppresses Enterobacteriaceae more efficiently than spontaneous fermentation of carrots. Food Microbiol..

[10] Zheng J., Wittouck S., Salvetti E., Franz C., Harris H.M.B., Mattarelli P., O’Toole P.W., Pot B., Vandamme P., Walter J. (2020). A taxonomic note on the genus *Lactobacillus*: Description of 23 novel genera, emended description of the genus *Lactobacillus Beijerinck* 1901, and union of *Lactobacillaceae* and *Leuconostocaceae*. Int. J. Syst. Evol. Microbiol..

[11] Paulino do Nascimento L.C., Lacerda D.C., Ferreira D.J.S., de Souza E.L., de Brito Alves J.L. (2022). Limosilactobacillus fermentum, Current Evidence on the Antioxidant Properties and Opportunities to be Exploited as a Probiotic Microorganism. Probiotics Antimicrob. Proteins.

[12] Marco M.L., Heeney D., Binda S., Cifelli C.J., Cotter P.D., Foligné B., Gänzle M., Kort R., Pasin G., Pihlanto A. (2017). Health benefits of fermented foods: Microbiota and beyond. Curr. Opin. Biotechnol..

[13] Anaya-Esparza L.M., Mora Z.V.-d.l., Vázquez-Paulino O., Ascencio F., Villarruel-López A. (2021). Bell peppers (*Capsicum annum* L.) losses and wastes: Source for food and pharmaceutical applications. Molecules.

[14] Li Y., Peng Y., Shen Y., Zhang Y., Liu L., Yang X. (2022). Dietary polyphenols: Regulate the advanced glycation end products-RAGE axis and the microbiota-gut-brain axis to prevent neurodegenerative diseases. Crit. Rev. Food Sci. Nutr..

[15] Guiné R.P.F., Barroca M.J. (2011). Effect of Drying on the Textural Attributes of Bell Pepper and Pumpkin. Dry. Technol..

[16] Hu X., Saravanakumar K., Jin T., Wang M.H. (2021). Effects of yellow and red bell pepper (paprika) extracts on pathogenic microorganisms, cancerous cells and inhibition of survivin. J. Food Sci. Technol..

[17] Hallmann E., Marszałek K., Lipowski J., Jasińska U., Kazimierczak R., Średnicka-Tober D., Rembiałkowska E. (2019). Polyphenols and carotenoids in pickled bell pepper from organic and conventional production. Food Chem..

[18] Blanco-Ríos A.K., Medina-Juárez L.Á., González-Aguilar G.A., Gámez-Meza N. (2013). Antioxidant activity of the phenolic and oily fractions of different sweet bell peppers. J. Mex. Chem. Soc..

[19] Thuphairo K., Sornchan P., Suttisansanee U. (2019). Bioactive compounds, antioxidant activity and inhibition of key enzymes relevant to Alzheimer’s disease from sweet pepper (*Capsicum annuum*) extracts. Prev. Nutr. Food Sci..

[20] Ropelewska E., Sabanci K., Aslan M.F. (2022). The Changes in Bell Pepper Flesh as a Result of Lacto-Fermentation Evaluated Using Image Features and Machine Learning. Foods.

[21] Althaus B., Blanke M. (2020). Non-destructive, opto-electronic determination of the freshness and shrivel of Bell pepper fruits. J. Imaging.

[22] Howard L., Talcott S., Brenes C., Villalon B. (2000). Changes in phytochemical and antioxidant activity of selected pepper cultivars (*Capsicum species*) as influenced by maturity. J. Agric. Food Chem..

[23] Montet D., Ray R.C., Zakhia-Rozis N., Ray R.C., Montet D. (2014). Lactic acid fermentation of vegetables and fruits. Microorganisms and Fermentation of Traditional Foods.

[24] Kim J.-H., Block D.E., Shoemaker S.P., Mills D.A. (2010). Conversion of rice straw to bio-based chemicals: An integrated process using *Lactobacillus brevis*. Appl. Microbiol. Biotechnol..

[25] Rybak K., Wiktor A., Witrowa-Rajchert D., Parniakov O., Nowacka M. (2020). The Effect of Traditional and Non-Thermal Treatments on the Bioactive Compounds and Sugars Content of Red Bell Pepper. Molecules.

[26] USDA5 Peppers, Bell, Green, Raw. https://fdc.nal.usda.gov/fdc-app.html#/food-details/2258588/nutrients.

[27] Verce M., De Vuyst L., Weckx S. (2020). Comparative genomics of *Lactobacillus fermentum* suggests a free-living lifestyle of this lactic acid bacterial species. Food Microbiol..

[28] Zhang Y., Vadlani P.V. (2015). Lactic acid production from biomass-derived sugars via co-fermentation of *Lactobacillus brevis* and *Lactobacillus plantarum*. J. Biosci. Bioeng..

[29] Fu W., Mathews A. (1999). Lactic acid production from lactose by *Lactobacillus plantarum*: Kinetic model and effects of pH, substrate, and oxygen. Biochem. Eng. J..

[30] Guiné R.P.F., Barroca M.J. (2012). Effect of drying treatments on texture and color of vegetables (pumpkin and green pepper). Food Bioprod. Process..

[31] Parniakov O., Bals O., Lebovka N., Vorobiev E. (2016). Effects of pulsed electric fields assisted osmotic dehydration on freezing-thawing and texture of apple tissue. J. Food Eng..

[32] Yang Z., Duan X., Yang J., Wang H., Liu F., Xu X., Pan S. (2022). Effects of high hydrostatic pressure and thermal treatment on texture properties of pickled kohlrabi. LWT—Food Sci. Technol..

[33] Zi Y., Zhixun S., Meiqi L., Xuetin Z., Hongbing R., Xiaosong H., Junjie Y. (2022). Effect of ripening and variety on the physiochemical quality and flavor of fermented Chinese chili pepper (*Paojiao*). Food Chem..

[34] Mapelli-Brahm P., Barba F.J., Remize F., Garcia C., Fessard A., Mousavi Khaneghah A., Sant’Ana A.S., Lorenzo J.M., Montesano D., Meléndez-Martínez A.J. (2020). The impact of fermentation processes on the production, retention and bioavailability of carotenoids: An overview. Trends Food Sci. Technol..

[35] Sano Y., Endo K., Tomo T., Noguchi T. (2015). Modified molecular interactions of the pheophytin and plastoquinone electron acceptors in photosystem II of chlorophyll d-containing Acaryochloris marina as revealed by FTIR spectroscopy. Photosynth. Res..

[36] Karcz D., Boroń B., Matwijczuk A., Furso J., Staroń J., Ratuszna A., Fiedor L. (2014). Lessons from Chlorophylls: Modifications of Porphyrinoids Towards Optimized Solar Energy Conversion. Molecules.

[37] Kang Y.-R., Park J., Jung S.K., Chang Y.H. (2018). Synthesis, characterization, and functional properties of chlorophylls, pheophytins, and Zn-pheophytins. Food Chem..

[38] Alberto M.R., Perera M.F., Arena M.E. (2013). Lactic acid fermentation of peppers. Food Nutr. Sci..

[39] Janiszewska-Turak E., Tracz K., Bielińska P., Rybak K., Pobiega K., Gniewosz M., Woźniak Ł., Gramza-Michałowska A. (2022). The Impact of the Fermentation Method on the Pigment Content in Pickled Beetroot and Red Bell Pepper Juices and Freeze-Dried Powders. Appl. Sci..

[40] Cierach M., Niedźwiedź J. (2014). Effects of three lighting intensities during display on discolouration of beef semitendinosus muscle. Eur. Food Res. Technol..

[41] Spínola V., Mendes B., Câmara J.S., Castilho P.C. (2012). An improved and fast UHPLC-PDA methodology for determination of L-ascorbic and dehydroascorbic acids in fruits and vegetables. Evaluation of degradation rate during storage. Anal. Bioanal. Chem..

[42] Nowacka M., Wiktor A., Anuszewska A., Dadan M., Rybak K., Witrowa-Rajchert D. (2019). The application of unconventional technologies as pulsed electric field, ultrasound and microwave-vacuum drying in the production of dried cranberry snacks. Ultrason. Sonochemistry.

[43] Schweiggert U., Kammerer D.R., Carle R., Schieber A. (2005). Characterization of carotenoids and carotenoid esters in red pepper pods (*Capsicum annuum* L.) by high-performance liquid chromatography/atmospheric pressure chemical ionization mass spectrometry. Rapid Commun. Mass Spectrom..

[44] Huang S., Hung C., Wu W., Chen B. (2008). Determination of chlorophylls and their derivatives in Gynostemma pentaphyllum Makino by liquid chromatography–mass spectrometry. J. Pharm. Biomed. Anal..

[45] Wiktor A., Chadzynska M., Rybak K., Dadan M., Witrowa-Rajchert D., Nowacka M. (2022). The Influence of Polyols on the Process Kinetics and Bioactive Substance Content in Osmotic Dehydrated Organic Strawberries. Molecules.

