# Supplementary material for: The Influence of Lactic Acid Fermentation on Selected Properties of Pickled Red, Yellow, and Green Bell Peppers
Source: Molecules. 2022 Dec 6;27(23):8637. doi: 10.3390/molecules27238637 (PMC9741357; doi:10.3390/molecules27238637)
Supplement: Supplementary file 1 [file molecules-27-08637-s001.zip › molecules-2063988-supplementary.pdf]

The Influence of the Lactic Acid Fermentation on selected properties of the Pickled Red, Yellow, and Green Bell Peppers

Emilia Janiszewska-Turak [1, *], Dorota Witrowa-Rajchert [1], Katarzyna Rybak [1], Joanna Rolof [1], Katarzyna Pobiega [2], Łukasz Woźniak [3] and Anna Gramza-Michałowska [4,]

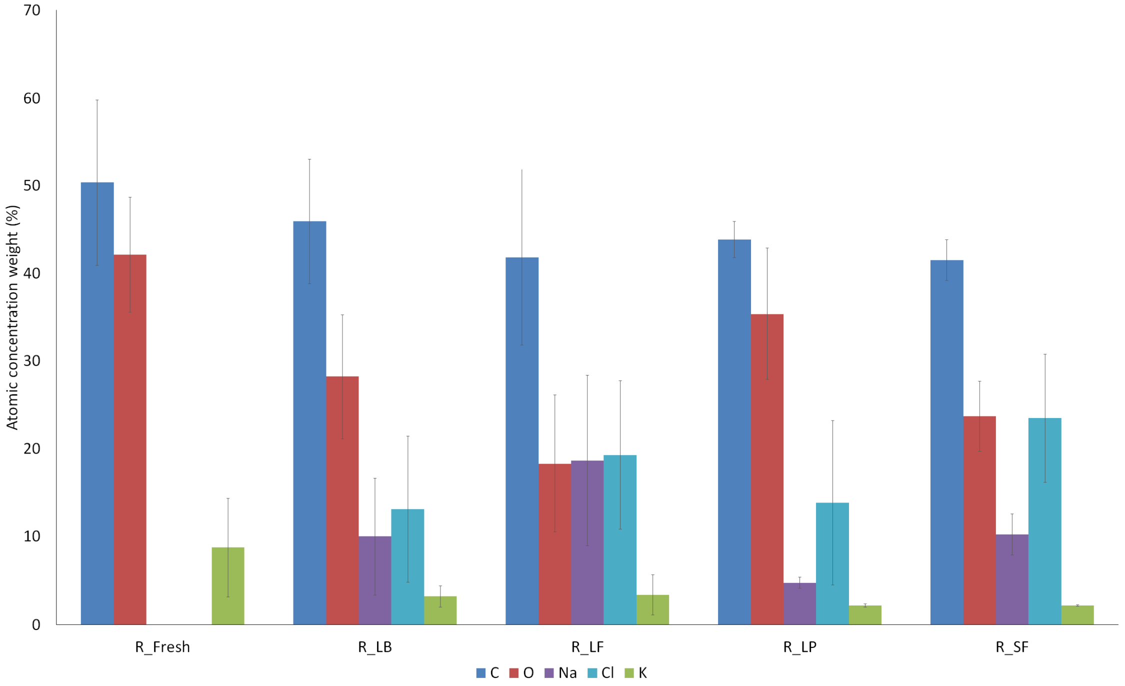

(**a**)

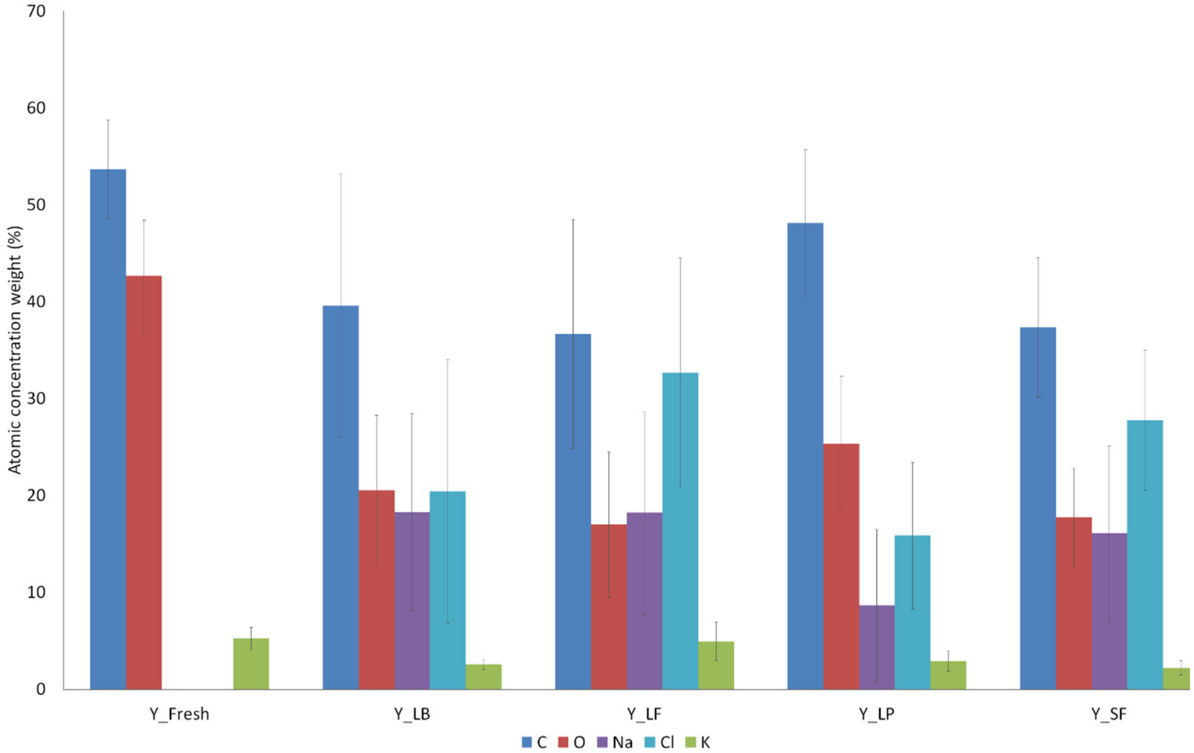

(**b**)

The Influence of the Lactic Acid Fermentation on selected properties of the Pickled Red, Yellow, and Green Bell Peppers

Emilia Janiszewska-Turak [1,*], Dorota Witrowa-Rajchert [1], Katarzyna Rybak [1], Joanna Rolof [1], Katarzyna Pobiega [2], Łukasz Woźniak [3] and Anna Gramza-Michałowska [4,]

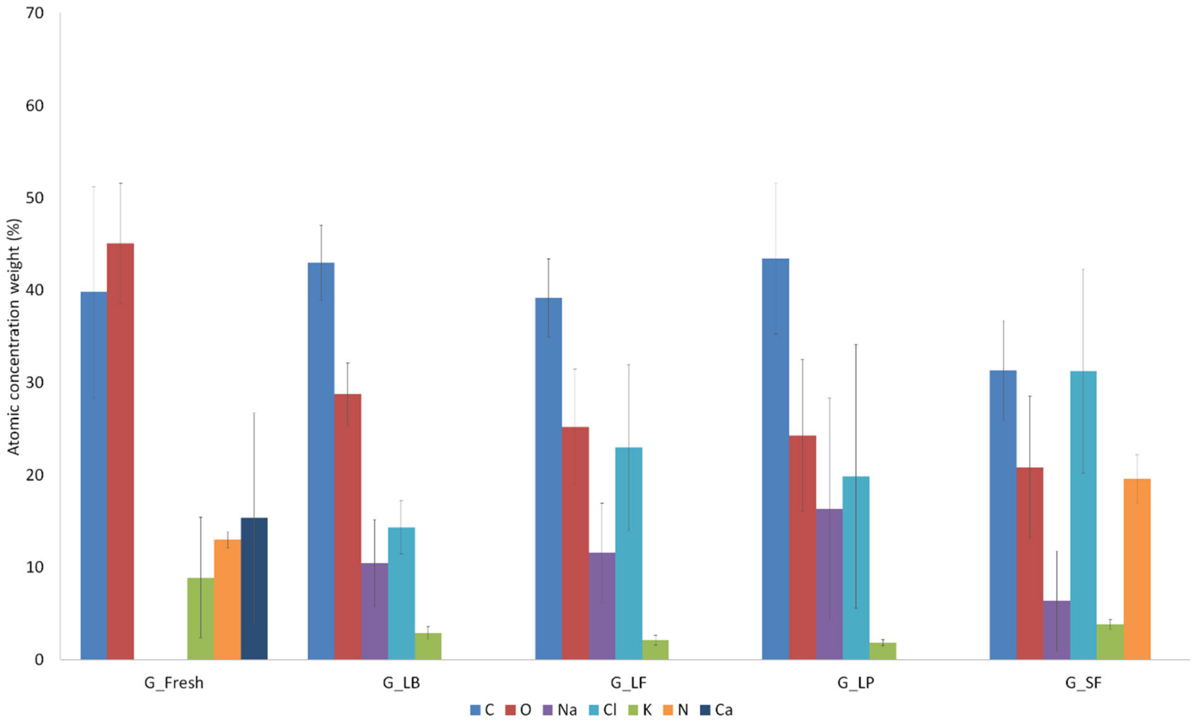

(**c**)

Figure S1. Results of SEM-EDS analysis for bell peppers (**a**) Red; (**b**) Yellow; (c) Green.

The Influence of the Lactic Acid Fermentation on selected properties of the Pickled Red, Yellow, and Green Bell Peppers

Emilia Janiszewska-Turak [1,*], Dorota Witrowa-Rajchert [1], Katarzyna Rybak [1], Joanna Rolof [1], Katarzyna Pobiega [2], Łukasz Woźniak [3] and Anna Gramza-Michałowska [4,]

Table S1. Compound identification parameters.

compound	Rt (min)	m/z
chlorophyll a	6.00	893.54
chlorophyll b	3.64	907.52
pheophytin a	9.83	871.57
pheophytin b	7.17	885.55
β-carotene	13.87	537.45
capsanthin	6.31	583.42
capsorubin	5.60	599.50
zeaxanthin	8.61	567.42
lutein	8.31	567.42
β-cryptoxanthin	7.67	551.50
capsanthin-laurate	14.31	765.58
capsanthin-myristate	15.35	793.61
capsanthin-palmitate	16.36	821.68
zeaxanthin-laurate	16.63	751.52
zeaxanthin-myristate	17.59	779.59
zeaxanthin-palmitate	18.53	807.65
capsanthin-di-laurate	18.29	949.76
capsanthin-laurate-myristate	20.15	977.80
capsanthin-di-myristate	21.94	1005.83
capsanthin-laurate-palmitate	22.17	1005.83
capsanthin-myristate-palmitate	23.54	1033.86
capsanthin-di-palmitate	25.02	1061.89
zeaxanthin-di-laurate	22.14	933.81
zeaxanthin-laurate-myristate	23.75	961.84
zeaxanthin-di-myristate	25.26	989.87
zeaxanthin-laurate-palmitate	25.63	989.87
zeaxanthin-myristate-palmitate	27.33	1017.86
zeaxanthin-di-palmitate	28.34	1045.89
